# Efficacy of Empiric Contact Precautions for Patients from High Risk Facilities

**DOI:** 10.1017/ash.2024.327

**Published:** 2024-09-16

**Authors:** Kavitha Prabaker, Dan Uslan, Annabelle De St. Maurice, Shaunte Walton, Vanessa Lewis, Anjali Bisht, Sebora Turay, Urvashi Parti, Ricardo Ison, Donna Wellbaum, Yvonne Mugford

**Affiliations:** UCLA Santa Monica; UCLA Health; UCLA David Geffen School of Medicine; Department of Clinical Epidemiology and Infection Prevention, UCLA Health, Los Angeles, CA, USA; Keck Medicine of USC

## Abstract

**Background:** Infection prevention surveillance revealed that patients admitted from two specific long term care facilities comprised the majority of multi-drug resistant organisms (MDRO) and scabies cases at our institution. Current practices include performing active surveillance for Candida auris and methicillin-resistant Staphylococcus aureus (MRSA) for specific high-risk patients, as surveillance for all MDROs and scabies is impractical. We therefore sought to create an admission screening process to efficiently identify patients from high-risk facilities (HRFs) and place them in pre-emptive contact precautions upon admission. **Methods:** Patients admitted from HRFs were identified on admission as part of the initial nursing assessment. For any positive responses, nursing received a Best Practice Advisory to place the patient in contact precautions and patient placement received an alert that the patient would require a private room. Infection Preventionists reviewed a report of all patients who screened positive and added a “High Risk Facility” banner to the chart. This banner remained for the duration of hospitalization and for every subsequent readmission and outpatient visit. We reviewed the electronic medical records of all patients with a HRF banner placed from March 8, 2023 to September 15, 2023 and abstracted data regarding the presence of scabies or any of the following MDROs before and after placement of the banner: C. auris, carbapenem-resistant enterobacterales (CRE), MRSA, vancomycin-resistant Enterococcus (VRE), carbapenem-resistant Acinetobacter, and MDR Pseudomonas. **Results:** Of the 93 patients who had a HRF banner added during the study period, 31 (33.33%) were already known to have MDRO colonization at the time of admission to our facility. Thirty-three of the remaining 62 patients (53.22%) without known MDRO colonization were subsequently found to have MDRO colonization/infection or scabies infestation that may have required contact precautions during their index admission or a subsequent admission. This included 14 patients with C. auris, 2 with CRE, 3 with MDR Pseudomonas, 12 with MRSA, 12 with carbapenem-resistant Acinetobacter, and 2 with VRE. Patients were admitted for a median of 9 days before their diagnosis, and 36 of the 93 patients (38.71%) were re-admitted to our hospital during the study period. **Conclusion:** We found that empiric contact precautions based solely on exposure to specific HRFs facilitated earlier isolation by a median of 9 days. This approach should be considered in acute care hospitals with a high proportion of admissions from HRFs, especially when active and passive surveillance for MDROs is limited.

